# Impact of different obstetric interventions and types of delivery on breastfeeding: a nationwide cross-sectional survey of Hungarian women

**DOI:** 10.1186/s12884-024-06666-x

**Published:** 2024-07-11

**Authors:** Anita Hulman, Annamária Pakai, Tímea Csákvári, Katalin Varga

**Affiliations:** 1 Doctoral School of Health Sciences, Faculty of Health Sciences, University of Pécs, 4 Vörösmarty str, Pécs, H-7621 Hungary; 2 Institute of Emergency Care, Pedagogy of Health and Nursing Sciences, University of Pécs, 4 Vörösmarty str, Pécs, H-7621 Hungary; 3 Department of Health Economics and Health Care Management, Institute of Health Insurance, Faculty of Health Sciences, University of Pécs, 33 Landorhegyi str, Zalaegerszeg, H-8900 Hungary; 4 Department of Affective Psychology, Institute of Psychology, ELTE Eötvös Loránd University, 46 Izabella str, Budapest, H-1064 Hungary

**Keywords:** Obstetric interventions, Synthetic oxytocin, Analgesia, Caesarean section, Breastfeeding

## Abstract

**Background:**

We assessed the effect of different obstetric interventions and types of delivery on breastfeeding.

**Methods:**

A quantitative, cross-sectional study was carried out using an online questionnaire. Data collection was performed in 2021 in Hungary. We included biological mothers who had raised their at least 5-year-old child(ren) at home (*N* = 2,008). The questionnaire was completed anonymously and voluntarily. In addition to sociodemographic data (age, residence, marital status, education, occupation, income status, number of biological children, and anthropometric questions about the child and the mother), we asked about the interventions used during childbirth, and the different ways of infant feeding used. Statistical analysis was carried out using Microsoft Excel 365 and SPSS 25.0. Descriptive statistics, two-sample t tests, χ^2^ tests and ANOVA were used to analyse the relationship or differences between the variables (*p* < 0,05).

**Results:**

We found that in deliveries where synthetic oxytocin was used for both induction and acceleration, there was a higher incidence of emergency cesarean section. However, the occurrence of vaginal deliveries was significantly higher in cases where oxytocin administration was solely for the purpose of accelerating labour (*p* < 0.001).Mothers who received synthetic oxytocin also received analgesics (*p* < 0.001). Women giving birth naturally who used oxytocin had a lower success of breastfeeding their newborn in the delivery room (*p* < 0.001). Children of mothers who received obstetric analgesia had a higher rate of complementary formula feeding (*p* < 0.001). Newborns born naturally had a higher rate of breastfeeding in the delivery room (*p* < 0.001) and less formula feeding in the hospital (*p* < 0.001). Infants who were breastfed in the delivery room were breastfed for longer periods (*p* < 0.001). Exclusive breastfeeding up to six months was longer for infants born naturally (*p* = 0.005), but there was no difference in the length of breastfeeding (*p* = 0.081).

**Conclusions:**

Obstetric interventions may increase the need for further interventions and have a negative impact on early or successful breastfeeding.

**Trial registration:**

Not relevant.

## Background

The 2018 World Health Organization (WHO) recommendation “Intrapartum care for a positive childbirth experience” summarizes existing and new recommendations on interventions for childbirth, labour and newborn care. The guidelines aim to make childbirth not only a safe but also a positive experience for women and their families. Furthermore, significant cost savings can be achieved by reducing unnecessary interventions during labour and birth when following the guidelines [1]. In Hungary, the 2019 guideline of the Ministry of Human Resources on the ‘Professional Guidelines for Family- Friendly Principles Maternity and Neonatal Care’ is an attempt to improve the quality of care, prepared by adapting the recommendations of the WHO’s abovementioned recommendation and NICE’s “Intrapartum care for healthy women and babies” guidelines from 2018, accounting for the specificities of the Hungarian health care system [2].

Since 2014, the WHO has recommended exclusive breastfeeding until 6 months of age, followed by breastfeeding until at least 2 years of age, with appropriate complementary feeding, to promote and support breastfeeding [3]. The exclusive breastfeeding rate is the lowest in the European Region (25%) [4]. According to Hungarian data collected according to the WHO definition, only 34.4% of newborns discharged from the hospital were exclusively breastfed until six months of age in 2019 [5].

Breastfeeding is, however, affected by many internal and external factors. An essential component of certain reproductive processes, such as sexuality, childbirth and breastfeeding, is oxytocin, which is produced in the hypothalamus and fine-tunes both peripheral and central events. It exerts its central effects within the brain as a neurotransmitter and, in addition to emotions, tunes social relationships and cognitive functions and influences the pathophysiology of neuropsychiatric diseases. In the periphery, it acts as a hormone to promote sexual activity, fertilization, childbirth and lactation [6]. Certain obstetric interventions, such as the use of exogenous (synthetic) oxytocin, epidural anaesthesia or caesarean section, can adversely affect the natural release of oxytocin. In a systematic review, Varga et al. reported a growing number of studies demonstrating that synthetic oxytocin used to induce and/or accelerate labour has an effect on the natural oxytocin system and may have a potential proximal or distal effect on the mother and child and their relationship [6]. Reviews indicate that nonspontaneous births have a higher rate of adverse outcomes. Despite these findings, exogenous oxytocin utilization for labour induction and/or acceleration is increasing in developed countries. In the United States of America, the frequency of exogenous oxytocin utilization is estimated at 50–60%, which, if routine postpartum intramuscular injection of synthetic oxytocin is included, essentially implies that the proportion of women who use synthetic oxytocin during labour is in the majority [6]. Cadwell and Brimdyr present the disadvantages of using synthetic oxytocin for breastfeeding [7]. Negative effects include reduced maternal endogenous oxytocin, increased risk of negative neonatal outcomes and reduced neonatal rest in the first hour, which adversely affects neonatal memory consolidation. In addition to the signs of hunger among newborns, the suckling reflexes may also be reduced. Newborns born to mothers administered synthetic oxytocin in developing countries are less likely to initiate suckling in the first hour of life. This is noteworthy given estimates suggesting that 22% of neonatal deaths could be prevented if breastfeeding were established within the initial hour of life [8].

In addition to synthetic oxytocin, the use of obstetric analgesics and anaesthesia, such as epidural anaesthesia (EDA), is also common. Their active agents are transferred across the placenta into the foetus, in many cases adversely affecting the outcome of delivery, the postpartum days and the infant’s ability to suckle in the postpartum period [9]. In addition to blocking the nerves that mediate pain, epidural anaesthesia also has a similar effect on the nerves that mediate the Ferguson reflex. This may have adverse effects on labour, breastfeeding and psycho-affective processes due to the reduced peripheral and central effects of oxytocin on labour [10]. Uterine activity is increased compared to natural activity when using synthetic oxytocin, which also increases the rate of fetal hypoxia and can lead to caesarean Sect. [11]. The number of deliveries ending in caesarean section was twice as high for deliveries with synthetic oxytocin compared to normal vaginal deliveries.

The combination of synthetic oxytocin and EDA used to induce and/or accelerate labour may cause cardiac arrhythmias in the foetus, which may justify a caesarean Sect. [12]. Lieberman et al. described that adverse fetal positioning was common in EDA, leading to prolonged labour, and found an association between the use of epidural anaesthesia and an increased incidence of caesarean section. In addition, poorer suckling skills were recorded in infants born with epidural anaesthesia (Infant Breastfeeding Assessment Tool) than in infants born without such intervention [13]. Tan et al. analysed the prevalence of breastfeeding among mothers undergoing epidural anaesthesia and found that 20% of mothers stopped breastfeeding between 5 and 9 weeks after birth (mean = 34 days), stating insufficient amounts of breast milk as the most common reason [14]. Raihana et al. [15] and Getaneh et al. [16] describe in their studies that children born by caesarean section have delayed initiation of breastfeeding. A literature review published in 2021, as well as Wu et al., showed a correlation between caesarean section and the onset and duration of breastfeeding [17, 18]. Compared to natural birth, caesarean section may delay the start and shorten the duration of exclusive breastfeeding. However, the planned caesarean section is one of the most critical factors affecting breastfeeding. An unfavourable trend of increased caesarean sections is shown in Hungary, from 33.0% in 2010 to 40.2% by 2017.

Beake et al. mentioned in their literature review that breastfeeding rates after caesarean section are lower than those after vaginal delivery. The caesarean section rate is 25% in many high-income countries and between 14% and 33% in other countries. In addition, the authors concluded that the rate of initiation of breastfeeding was significantly lower after caesarean section. The rates of exclusive breastfeeding up to 6 months of age and for partial breastfeeding were also lower among women who had a caesarean section (whether planned or unplanned) compared with vaginal delivery [19].

Madison et al. also showed that the use of synthetic oxytocin, epidural anaesthesia and caesarean section can increase the time a newborn is fed with complementary formula in a hospital and shorten the time of exclusive breastfeeding by 3 months after birth [20].

Our study aims to assess the association of obstetric interventions, in particular synthetic oxytocin, obstetric analgesia and type of delivery, with breastfeeding in a Hungarian sample. Most women give birth in healthcare facilities, but in many cases, the quality of care is suboptimal. In some cases, too little intervention is done too early, while in other circumstances, too much is done too late, which continues to hinder the achievement of desired health outcomes such as successful breastfeeding. This paper explores the relationship between obstetric interventions and breastfeeding. Our research aims to contribute to the optimization of the interventions under study to achieve breastfeeding as early and for as long as possible. We also aim to reduce the rate of obstetric interventions and thereby indirectly improve breastfeeding rates.

## Methods

### Type of study, objective

Our quantitative, cross-sectional study analysed the circumstances and events of childbirth, highlighting the occurrence of obstetric interventions, the type of delivery and the characteristics of infant feeding, to identify possible associations that could reduce the extent of interventions that negatively affect breastfeeding.

### Study design and participants

Our quantitative, cross-sectional study was conducted online with a nonrandom, targeted sampling method between 26 March and 18 July 2021. The inclusion criteria were met by biological mothers who had at least one child born alive after the 37th week of gestation and raised in their care and were no more than 5 years old (60 months) at the time of completing the questionnaire. We excluded those not completing the questionnaire for their biological child, those who were pregnant with their first child at the time of the study, and mothers who completed the mandatory questions incompletely or inaccurately, potentially skewing the results. In addition, the presence of any congenital or acquired physical or mental illness of the child or mother that made breastfeeding impractical was also an exclusion criterion. In total, 2505 people completed the questionnaire, of whom 2008 people met the inclusion criteria, creating the sample population.

We posted a link to a Google Form as the questionnaire on social platforms created for Hungarian pregnant women and mothers on several occasions. The questionnaire was completed anonymously and voluntarily. The questionnaire did not contain any questions that would have revealed the identity of the respondent. If the participant did not wish to complete the questionnaire or did not wish to answer the questions, they were given the option to stop at any time, and their answers were not submitted. All those who completed the questionnaire and finalized and submitted their answers by clicking on the ‘submit’ button agreed to participate in the research. The responses were sent exclusively to the Google account hulmananita@gmail.com managed by the first author (Anita Hulman). For responses received, only the date of submission was visible to the author, and the email address of the respondent was not recorded.

### Data collection

Prior to our research, a systematic literature search was carried out for several months in PubMed, Medline, Google Scholar, Wiley, Scopus, and BMC. Relevant literature was categorized according to various criteria and then further classified according to logical and content criteria. For our research, we opted for a self-administered questionnaire survey. After several weeks of review work, we prioritized the themes and defined and formulated the questions in accordance with our research objectives. We then conducted a PILOT survey with 6 respondents to identify and correct any errors based on their comments. The completed questionnaire and the research draft were approved by the Regional Committee of Research Ethics at the Markusovszky Hospital in Szombathely, Hungary. The planned scientific work was considered appropriate and up-to-date, and permission to start was granted in strict compliance with data protection rules (Chairperson: Prof Béla Márkus, chief physician, scientific advisor; study number: 13/2021). The survey was then conducted online for 4 months, considering all the relevant guidelines and regulations.

The main reason for collecting data online was to create a large sample from a broad area. The questionnaire consisted of a total of 74 questions. Closed and semi-open questions were formulated to allow for the formation of an independent opinion.

In addition to sociodemographic data (age, residence, marital status, education, occupation, income status, number of biological children, and anthropometric questions about the child and the mother), we asked about the interventions used during childbirth and the circumstances relevant to our research. We also collected data on breastfeeding and other characteristics of infant feeding. The prevalences of the obstetric interventions within the study population are illustrated in Table 1. In Table 2, the obstetric interventions studied are further analysed according to the mode of delivery in three categories: vaginal birth, elective caesarean section, and emergency caesarean section.

**Table 1:** Foods to be given according to the feeding method

	Breast milk	Boiled or baby water	Unflavoured tea	Formula	Complementary feeding
Exclusive breastfeeding	✓	**×**	**×**	**×**	**×**
Breastfeeding	✓	✓	✓	**×**	**×**
Mixed feeding	✓	✓	✓	✓	**×**
Formula feeding	**×**	✓	✓	✓	**×**
Breast- and complementary feeding	✓	✓	✓	**×**	✓
Formula and complementary feeding	**×**	✓	✓	✓	✓

**Table 2:** Frequencies of different obstetric interventions by type of delivery (*n* = 2008)

	Vaginal delivery (*n* = 1274)	Elective caesarean section (*n* = 253)	Emergency caesarean section (*n* = 481)	χ^2^(2)	*p*
Induced	263 (20.6%)	58 (22.9%)	**206 (42.8%)**	90.4	< 0.001
Not induced	1011 (79.4%)	195 (77.1%)	275 (57.2%)		
Accelerated	695 (54.6%)	19 (7.5%)	253 (52.6%)	192.1	< 0.001
Not accelerated	579 (45.4%)	**234 (92.5%)**	228 (47.4%)		
Both induced and accelerated	203 (15.9%)	15 (5.9%)	**144 (29.9%)**	75.0	< 0.001
Neither induced nor accelerated	**1071 (84.1%)**	**238 (94.1%)**	337 (70.1%)		
Analgesia (spinal, EDA)	185 (14.5%)	201 (79.4%)	**312 (64.9%)**	645.1	< 0.001
No use of analgesia	**1089 (85.5%)**	52 (20.6%)	169 (35.1%)		
Perineotomy	**815 (64%)**	0 (0%)	3 (0.6%)	779.4	< 0.001
No use of perineotomy	459 (36%)	253 (100%)	478 (99.4%)		
Amniotomy	**711 (55.8%)**	16 (6.3%)	190 (39.5%)	218.0	< 0.001
No use of amniotomy	563 (44.2%)	**237 (93.7%)**	291 (60.5%)		
Use of vacuum extractor or forceps	**69 (5.4%)**	1 (0.4%)	2 (0.4%)	33.8	< 0.001
No use of vacuum extractor or forceps	1205 (94.6%)	252 (99.6%)	479 (99.6%)		
General anaesthesia	13 (1%)	19 (7.5%)	**54 (11.2%)**	96.077	< 0.001
No use of general anaesthesia	1261 (99%)	234 (92.5%)	427 (88.8%)		
Narcotic gas	**153 (12%)**	12 (4.7%)	**62 (12.9%)**	12.7	0.002
No use of narcotic gas	1121 (88%)	241 (95.3%)	419 (87.1%)		

Regarding the method of feeding, mothers were asked to select the feeding method they used during the first 6 months of the infant’s life.

An *exclusively breastfed* child was an infant who did not receive any fluids other than breast milk - water, tea or formula.

*Breastfeeding* included natural breastfeeding and breast milk given from a bottle or any other assistive device, such as a Swedish drinking cup or syringe. We also included those who received, in addition to breast milk, a nonenergy-providing liquid - boiled or baby water, unflavoured tea - for the body.

*Mixed fed* infants received breast milk, formula and nonbreast milk, and nonenergy liquids such as boiled or baby water, unflavoured tea from a bottle or with an assistive device.

*Formula-fed* infants were fed with infant formula or were given a nonenergy-providing liquid - boiled or baby water, water, unflavoured tea - in addition.

*Breast- and complementary feeding* involve infants receiving breast milk alongside conventional foods, excluding formula.

Finally, when babies received only infant formula and conventional foods, they were classified as *formula and complementary feeding*.

### Statistical analysis

Statistical analysis was carried out using Microsoft Excel 365 and SPSS 25.0. Descriptive statistics were calculated to characterize the sample (absolute and relative frequencies, mean, standard deviation, minimum and maximum values). Two-sample t tests, χ^2^ tests and ANOVA were used to analyse the relationship or differences between the variables. The level of significance was defined at *p* < 0.05 [21].

## Results

### Sample characteristics

The mean age of the sample was 31.43 years (min 18 years, max 55 years, SD = 5.05). A total of 73.3% of respondents (*n* = 1472/2008) completed the questionnaire for their first child, 20.4% (*n* = 409/2008) for their second child and 6.3% (*n* = 126/2008) for their third child. A total of 36.7% (*n* = 737/2008) of mothers gave birth at term, and 63% (*n* = 1365/2008) gave birth beyond term and 0.03% (*n* = 6/2008) could not remember.

### Relationship between mode of delivery and breastfeeding

A total of 63.4% (*n* = 1274/2008) of the participants gave birth naturally, 24.0% (*n* = 481/2008) by emergency caesarean section and 12.6% (*n* = 253/2008) by planned caesarean section.

Significant differences were found between the induction of labour and different modes of delivery. The highest proportion of induction of labour was among parents who had an emergency caesarean section, at almost 50%, while in cases where no induction of labour was performed, most mothers gave birth naturally or by planned caesarean section (Table 2).

There is a significant difference between the type of delivery and the acceleration of labour with synthetic oxytocin. Accelerated delivery was least common in those who had a planned caesarean section, at less than 10%, while exogenous oxytocin acceleration occurred in more than half of those who delivered naturally and in more than half of those who had an emergency caesarean section. The incidence of natural births was found to be significantly higher in those who had an augmentation of labour for quicker delivery. The highest rates of both induction and augmentation were among mothers who had an emergency caesarean section. (Table 2)

There was a significant difference in the use of analgesics according to the type of delivery. Less than 15% of those who gave birth naturally and almost two-thirds of those who gave birth by emergency caesarean section received analgesics. (Table 2)

There was a significant difference in the use of medication for analgesia between those who received both induction and acceleration and those who did not (*p* < 0.001). A total of 49.7% (*n* = 180/362) of those who received both induction and acceleration received medication for analgesia, while only 31.5% (*n* = 518/1644) of those who did not receive both induction and acceleration received medication for analgesia.

There was a significant difference in the use of analgesics between mothers who received and those who did not receive exogenous oxytocin therapy (*p* < 0.001). 20% of those who received exogenous oxytocin therapy (*n* = 151/755) also received medication for pain relief. In contrast, a third as many patients who did not receive synthetic oxytocin, only 6.4% (*n* = 33/516), also received medication for pain.

### Relationship between mode of delivery and the conditions of obstetric and newborn care

There was a significant difference between the types of delivery according to breastfeeding in the delivery room (*p* < 0.001). Women who gave birth naturally had a significantly higher success of breastfeeding their babies in the 2 h (golden hour) they spent in the delivery room compared to those who delivered by caesarean section. A total of 76.3% of mothers who gave birth vaginally (*n* = 972/1274), compared with only 35.2% of mothers who had a planned caesarean section (*n* = 89/253) and a similar proportion of mothers who had an emergency caesarean section (*n* = 147/481), reported breastfeeding their baby in the delivery room.

A significantly higher proportion of mothers without exogenous oxytocin utilization breastfed their babies in the delivery room (*p* < 0.001). A total of 81.9% (*n* = 425/519) of mothers who did not receive exogenous oxytocin during delivery and 72.5% (*n* = 547/754) of mothers who did reported successful breastfeeding during their delivery room stay.

There was a significant difference in the need for complementary formula feeding within a hospital between newborns of mothers who did and did not receive analgesics (*p* < 0.001). Newborn infants of mothers who received medication for pain during delivery accounted for 55.3% (*n* = 365/660) of the total number of infants, while only 40.9% (*n* = 509/1244) of newborns of mothers who did not receive analgesics received complementary formula in the hospital.

In addition, there was a significant difference in the prevalence of formula feeding during hospitalization by mode of delivery (*p* < 0.001). A total of 61.3% (*n* = 751/1225) of babies born naturally did not need formula, while 61.7% (*n* = 271/439) of those born by emergency caesarean section received supplementary feeding. Out of the babies born by planned caesarean section, slightly fewer, 53.5% (*n* = 129/241), received formula supplementation during their hospital stay.

Exclusive breastfeeding up to six months of age was achieved by more than two-thirds of mothers who gave birth naturally, while the proportion of infants born by caesarean section was not much lower. (Table 3)

**Table 3:** Comparison of some feeding methods up to six months of age by the type of delivery

	Vaginal delivery (*n* = 1274)	Elective caesarean section (*n* = 253)	Emergency caesarean section (*n* = 481)	χ^2^(2)	*p*
Exclusively breastfed	**883 (69.3%)**	156 (61.7%)	307 (63.8%)	8.5	0.014
Other	391 (30.7%)	97 (38.3%)	174 (36.2%)		
Breastfed	**770 (60.4%)**	123 (48.6%)	**267 (55.5%)**	13.4	< 0.001
Other	504 (39.6%)	130 (51.4%)	214 (44.5%)		
Breastfed with complementary formula	234 (18.4%)	**66 (26.1%)**	99 (20.6%)	8.1	0.017
Other	1040 (81.6%)	187 (73.9%)	382 (79.4%)		
Formula (from bottle or another device)	437 (34.3%)	**125 (49.4%)**	198 (41.2%)	23.4	< 0.001
Other	837 (65.7%)	128 (50.6%)	283 (58.8%)		
Breastfeeding and complementary feeding	**166 (13%)**	20 (7.9%)	47 (9.8%)	7.5	0.024
Other	1108 (87%)	233 (92.1%)	434 (90.2%)		
Breastfeeding and complementary feeding	**84 (6.6%)**	29 (11.5%)	57 (11.9%)	15.8	< 0.001
Other	1190 (93.4%)	224 (88.5%)	424 (88.1%)		

* Other: any other type of feeding in the table that is not included in the given row

There is a significant difference in breastfeeding in the first six months of an infant’s life based on the type of birth. Breastfeeding was found to be highest for mothers who delivered naturally, at nearly 60%, while for parents who had a planned caesarean section, it was less than 50%. (Table 3)

One-fifth of mothers who gave birth naturally and one-fifth of mothers who had an emergency caesarean section used mixed feeding, while a slightly higher proportion of parents who had a planned caesarean section mixed fed their babies for the first 6 months. (Table 3)

We found significant differences between the mode of delivery in accordance with formula feeding. The largest proportion of babies born by planned caesarean section (nearly half) and just under a third of babies born naturally were formula-fed in the first 6 months of life (Table 3).

Only a few mothers started complementary feeding before the infant’s age of six months, but it was the most common among mothers delivering naturally, occurring in every one out of seven cases. Complementary feeding in addition to formula was prevalent among one in ten mothers who had a caesarean section and was less prevalent among mothers who gave birth naturally. (Table 3)

**Fig. 1:**
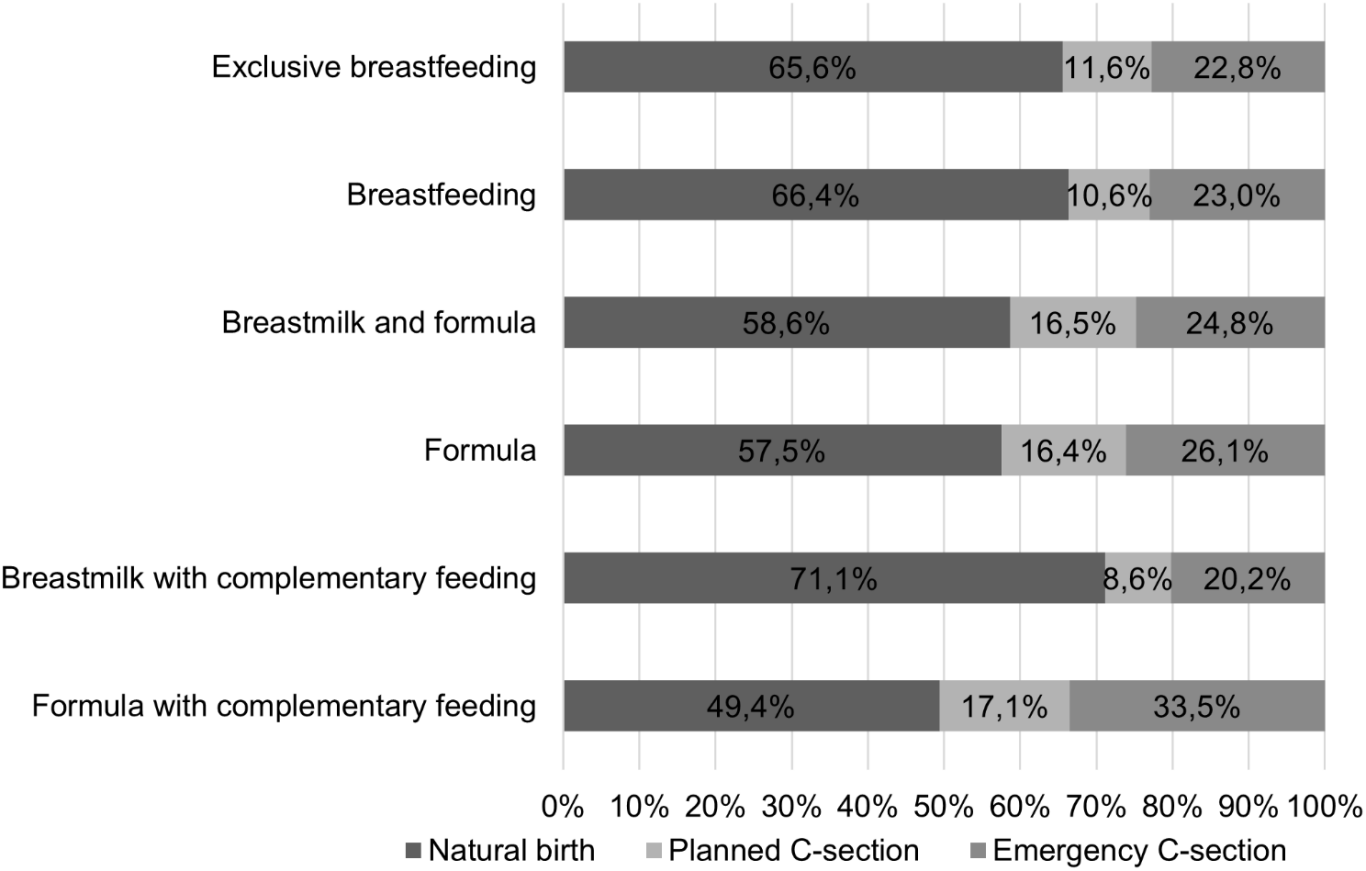
Characteristics of infants feeding up to 6 months of age (*n* = 2,008)

There was a significant difference in the duration of exclusive breast milk feeding between infants born in different ways (*p* = 0.005). We found that for those born naturally (*n* = 1077), the longest duration was 5.1 months on average (SD = 2.1, min = 0, max = 24), while for children born by planned caesarean section (*n* = 200), exclusive breastfeeding was achieved for the shortest period, averaging 4.5 months (SD = 2.3, min = 0, max = 11) (*p* = 0.005). The distribution of the proportions of different feeding modes by mode of delivery up to six months of age is shown in Fig. 1.

There was no significant difference in the duration of breast milk feeding regarding the type of delivery (*p* = 0.081). For those born naturally (*n* = 1246), the longest duration was 11.0 months on average (SD = 8.6, min = 0, max = 60); for those born by emergency caesarean section (*n* = 456), breastfeeding lasted on average 10.7 months (SD = 8.0, min = 0, max = 40); and for those born by planned caesarean section (*n* = 240), breastfeeding lasted shorter, on average 9.6 months (SD = 8.0, min = 0, max = 42) (*p* = 0.081).

However, newborns who were breastfed in the delivery room were breastfed for a significantly longer period than those whose first breastfeeding occurred later (*p* < 0.001). Babies who were breastfed in the delivery room were breastfed for an average of 11.4 months (SD = 8.5, min = 0, max = 60), while those who were not breastfed in the delivery room consumed breast milk for a shorter period, until 9.7 months (SD = 8.0, min = 0, max = 60).

## Discussion

Systematic literature reviews in recent years [10, 18] indicate that a higher proportion of non-spontaneous births that are could have adverse outcomes in terms of breastfeeding. Despite the scientific evidence, the use of exogenous oxytocin for routine induction or acceleration of labour is steadily increasing and is well reflected in our research findings.

It is fair to say that practical experience often contradicts the 2018 WHO recommendation ‘Intrapartum care for a positive childbirth experience’, which calls for avoiding unnecessary obstetric interventions, including the induction of labour [1]. However, the proportion of mothers who give birth without intervention is currently low. Beake mentions that caesarean section rates are rising rapidly, with many high-income countries reporting rates as high as 25%, while in other countries, it is between 14% and 33% [19]. In our study, the proportion of deliveries completed by caesarean section was 36.6%. Based on our results, similar to the publication by Varga et al. [6], we found that more than half of the mothers received synthetic oxytocin during delivery. More than a quarter used it to induce labour, while almost half used it to accelerate labour. Raba et al. explained that compared to the natural oxytocin produced in the body, the use of synthetic oxytocin induces more pronounced uterine activity, increasing the risk of oxygen deprivation and more often justifying a caesarean Sect. [11]. The authors found that the incidence of caesarean section was twice as high in cases where mothers received synthetic oxytocin during delivery. Buckley also highlights the link between synthetic oxytocin use and more frequent caesarean Sect. [12]. This relationship was also significant in our study (*p* < 0.001). A higher rate of caesarean section occurred in those who had induction of labour or who had both induction and acceleration of labour with synthetic oxytocin than in those who did not have induction or acceleration (*p* < 0.001). Uvnäs Moberg et al. and Tan et al. revealed the association between epidural anaesthesia and adverse labour, breastfeeding and psycho-affective processes [10]. Despite the data from this study, the use of medication for pain relief, particularly epidural anaesthesia (EDA), is still significant in obstetric units, and our results support this. One in three mothers in our study received obstetric analgesia. Lieberman et al. also point to an association between epidural anaesthesia and increased caesarean section prevalence [13], which is also reflected in our results, as two-thirds of those who received epidural or spinal anaesthesia delivered their babies by caesarean section.

It is important to highlight that the induction of labor before elective cesarean sections took place in the hospitals where the participants were treated. During planned cesarean sections, the use of synthetic oxytocin is also common to achieve adequate uterine tone. Initially, oxytocin bolus is administered for this purpose, followed by maintaining uterine contractions with a synthetic oxytocin infusion. This is supported by the research of Duffield et al. in 2017 [22].

The Infant Breastfeeding Assessment Tool reports that infants born with EDA have poorer suckling skills than newborns born without EDA. In addition, Madison et al. reported that newborns of mothers who received EDA achieved exclusive breastfeeding for a shorter period at 3 months of age compared to infants whose mothers did not receive EDA [20]. This finding is supported by our research, as we found a significant association between the use of analgesics during natural delivery and the supplementary formula feeding given to the newborn in the hospital. Newborns of mothers who received analgesics were significantly more likely to receive formula supplementation in the hospital (*p* < 0.001). Among the negative effects of synthetic oxytocin use on breastfeeding, Cadwell reports reduced neonatal hunger signals and sucking reflexes, which make babies less likely to suckle in the first hour of life [7]. Based on our results, mothers who gave birth naturally and who were given synthetic oxytocin during delivery had a lower rate of breastfeeding in the delivery room than those who were not given synthetic oxytocin (*p* < 0.001). Furthermore, Uvnäs Moberg et al. highlighted the negative impact of caesarean section on breastfeeding, as they found reduced milk production and a shorter duration of breastfeeding on the second postpartum day in mothers who delivered by Sect. [10]. In addition, Raihana et al. and Getaneh et al. report a positive association between caesarean section and delayed initiation of breastfeeding. A 2021 literature review also showed a link between caesarean section and the initiation and duration of breastfeeding; compared to natural childbirth, caesarean section can negatively affect the initiation and shorten the duration of exclusive breastfeeding [17]. We examined the association between the types of delivery and breastfeeding in the delivery room and found a significant difference, similar to the literature cited. Breastfeeding during golden hour (within two hours after birth) was twice as common in mothers delivered naturally than in those delivered by caesarean section (*p* < 0.001). Uvnäs Moberg reported that only 21% of mothers who delivered by caesarean section breastfed their babies in the delivery room [10]. We also found that one-third of mothers who had a caesarean section breastfed in the delivery room. Furthermore, similar to Madison et al., our results showed that a significantly higher proportion of infants born by caesarean section receive complementary formula feeding during their hospital stay than those born naturally (*p* < 0.001) (although even in the latter, the rate of formula feeding is still significant and contrary to recommendations) [20]. Contrary to the popular belief that caesarean section reduces milk production, this may be due to the difficulty of initiating ‘mothering’, the presumed less enjoyable nature of maternal care, the difficulty of mother-child synchrony and bonding, and a host of other psycho-affective factors. Beake et al. reported that breastfeeding initiation rates were significantly lower after caesarean Sect. [19]. We also expected to find a relationship between the type of delivery and the duration of breastfeeding. Babies born vaginally were exclusively breastfed for longer than babies born by caesarean section (*p* = 0.005); furthermore, they were breastfed for longer than babies born by caesarean section for the first six months (*p* = 0.014). Those born by caesarean section had significantly higher rates of formula feeding up to six months of age than those born naturally (*p* < 0.001). However, our research also reflects findings reported by Li et al., as babies who were born naturally are breastfed longer than those born by caesarean section, although not significantly (*p* = 0.081) [17].

In Hungary, even for cesarean section, the two-hours-long ‘golden hour’ should be ensured. A ministerial guideline specifies that ‘in the case of a cesarean section, even during the operation, within the ‘golden hour’ period, it is recommended to place the newborn on the mother’s chest with direct skin-to-skin contact. If this is not possible for medical reasons, it is recommended to place the newborn on the father’s (companion’s) chest with direct skin-to-skin contact and then transfer the baby to the mother as soon as it becomes feasible.’ This is possible and is implemented in some hospitals (referred to as humanized or natural cesarean section). However, it is far from being common. Hence, we emphasize the importance of additional support of mothers undergoing augmented labours and deliveries.

A limitation of the study is that complementary feeding may vary according to hospital protocol. The sample is not representative in any way, and the fact that the study was limited to mothers with access to the internet and the ability to use it may be a source of bias. All our data are self-reported with no exact knowledge on the dosage of analgesics and medication, which may introduce undetected errors and biases; however, there is no database in Hungary that collects obstetric interventions and postpartum events for the same delivery. Although online data collection does not provide a representative sample, it significantly increases the response rate while providing the opportunity for independent responses (for example, as opposed to data collected in an institution). Other sociocultural factors possibly affecting breastfeeding were not examined in this study.

## Conclusions

The use of synthetic oxytocin and obstetric analgesia, as well as the high rate of caesarean sections, can be observed in obstetric interventions. Based on our results, we can say that induction and both induction and acceleration of delivery ends in caesarean section occur more frequently. This cascade of interventions was also observed in our study, as the combined use of induction and acceleration of labour and analgesia was associated with an increase in the incidence of caesarean section, as almost half of induced deliveries end in it.

The type of birth may be related to, among other things, the onset and duration of breast and complementary feeding of the baby. The prevalence of formula feeding is higher in babies whose mothers have undergone epidural or spinal anaesthesia or caesarean section and in those who could not be breastfed in the delivery room. Children born naturally are the most likely, while children born by planned caesarean section are the least likely to be exclusively breastfed for the longest period. The type of delivery has a significant impact on the duration of breastfeeding until the infant’s 6 months of age, with a significant difference between those born naturally and those born by caesarean section. The highest proportion and longest duration of breastfeeding in the first six months of life is among babies born vaginally. In addition to the mode of delivery, early starting also has a significant role in breastfeeding, as those who were breastfed in the first two hours of life (golden hour) were significantly longer breastfed than those who were not breastfed in this time.

This study highlights the impact of possible negative effects of obstetric interventions on breastfeeding. Frequent intrapartum interventions could be associated with negative breastfeeding outcomes. This reinforces the importance of only intervening in the natural birth process when necessary. The support of mothers who undergo cesarean sections is particularly crucial to promote breastfeeding. However, it is essential to ensure mothers have the freedom to make decisions and consider their well-being both in obstetric care and breastfeeding. This plays a significant role, for example, in pain management during and after childbirth. Evidence supports that epidural pain relief can reduce postpartum depression (PPD). Eisenach et al. demonstrated that the severity of acute postpartum pain predicted a threefold increase in the risk of postpartum depression within 8 weeks [23]. It is undeniably important for mothers to receive adequate pain management to effectively care for their newborns. Various methods are known for pain relief treatment.

The U.S. Food and Drug Administration highlights the avoidance of opioids and nonsteroidal anti-inflammatory drugs (which are the cornerstones of managing moderate and severe acute pain) for breastfeeding women [24]. In addition to pharmacological methods, non-pharmacological pain management is also worth considering. [25].

To improve our obstetric and breastfeeding indicators, we recommend that the latest evidence-based research and recommendations (WHO) be known and applied in professional training and practice so that mothers at increased risk of early cessation of breastfeeding can be helped during and after their hospital stay [1, 2, 26–30]. Furthermore, additional studies are necessary to explore whether mothers undergoing planned cesarean sections are burdened by other risk factors concerning breastfeeding.

## Data Availability

The datasets used and/or analysed during the current study are available from the corresponding author upon reasonable request.

## Abbreviations

EDA: Epidural anaesthesia
WHO: World Health Organization
